# High-Efficient Excitation-Independent Blue Luminescent Carbon Dots

**DOI:** 10.1186/s11671-017-2137-2

**Published:** 2017-06-10

**Authors:** Hongzhen Liu, Xin Zhao, Fei Wang, Yunpeng Wang, Liang Guo, Jingjing Mei, Cancan Tian, Xiaotian Yang, Dongxu Zhao

**Affiliations:** 1 State Key Laboratory of Luminescence and Applications, Changchun Institute of Optics, Fine Mechanics and Physics, Chinese Academy of Sciences, No. 3888 Dongnanhu Road, Changchun, 130033 People’s Republic of China; 2 University of Chinese Academy of Science, Beijing, 100049 People’s Republic of China; 3 Jilin Provincial Key Laboratory of Architectural Electricity & Comprehensive Energy Saving, Jilin Jianzhu University, Changchun, 130118 People’s Republic of China

**Keywords:** Carbon dots, Luminescent, Concentration, Nanosized cluster

## Abstract

Blue luminescent carbon dots (CDs) were synthesized by the hydrothermal method. Blue-shifts of the maximum emission wavelength from 480 to 443 nm were observed when the concentration of CD solution decreased. The photoluminescence (PL) spectra of CDs at low concentration showed an excitation-independent behaviour, which is very different from the previous reports. Two different emitting mechanisms might work: the intrinsic luminescence from sp^2^-carbon networks can be responsible for the shorter wavelength part of emission (excitation-independent) at low concentration and the high polarity of nanosized clusters led to the excitation-dependent behaviour of the longer wavelength part at high concentration of CD solution. The photophysical property and concentration-dependent behaviour of the CDs offered new insights into CDs from the viewpoints of both experiments and mechanisms, which will promote diverse potential applications of CDs in the near future.

**Electronic supplementary material:**

The online version of this article (doi:10.1186/s11671-017-2137-2) contains supplementary material, which is available to authorized users.

## Background

Carbon dots, as a fluorescent material in the carbon nanomaterial family, have drawn increasing concerns in the past few years. Typically, a CD has a core of graphite or amorphous carbon framework, and the surface of which is coated with oxygen-containing groups, polymers and other species [1]. Meanwhile, CDs, which are not larger than 10 nm, have unique photophysical characteristics, such as high photostability, good biocompatibility, excellent optical properties and low environmental hazards [2, 3]. Inspired by these properties, the CDs possess various potential applications, such as drug delivery [4], fluorescent ink [5], sensors [6, 7], optoelectronics [8], photocatalysis [9, 10], and light-emitting devices [5, 11–13]. Up to now, various synthetic methods have been developed for the preparation of CDs, such as the electrochemical oxidation of graphite [9], the hydrothermal method [5, 10], and the microwave-assisted synthesis [14, 15].

One special property of CDs is the dependence of emission peak with excitation wavelength. Under different excitation wavelengths, CDs have different photoluminescence (PL) peaks from violet to red [10]. Many possible reasons have been reported to explain this phenomenon, such as size [9, 11], element doping [10, 14], solvent polarity [16], defects, surface states [17], surface groups [18, 19] or surface passivation [20]. However, the excitation-independent property of CDs is rarely observed.

Interestingly, we have found that by diluting CD solution with deionized water, the blue-shift of the maximum emission peak from 480 to 440 nm was observed. Also, the emission intensity of CDs became stronger with the decreasing concentration. The PL spectra showed an unchanging emission peak at 443 nm as the excitation wavelength varied, which is very different from the previous reports. The high polarity of nanosized cluster and the sp^2^-carbon networks can be responsible for these phenomena.

## Methods

### Reagents and Chemicals

Critric acid monohydrate (99.5%) was required from SCR (Shanghai, China), and Ethylendiamine was attained from Tianzheng reagent (Tianjin China). Deionized water was obtained from a water purifier water purification system with a resistivity 18.25 m Ω cm (Sichuan, China). All the chemicals were used as received without further purification or treatment.

### Preparation of Carbon Dots

CDs were prepared as follows: the citric acid (1.0507 g) and ethylendiamine (335 μL) were added into deionized water (10 mL). Then, the well-stirred solution was transferred to a Teflon-lined autoclave. The solution was heated to 150 °C for 5 h. After the reaction, the reactors were cooled to room temperature naturally. The colour of the prepared CD solution was yellowish. Before characterization, the CD solution was treated by the following methods: took 1 ml original CD solution and then diluted by 5–400 ml deionized water. The colour of the CD solution changed from yellow to colourless after the dilution.

### Characterization

The photoluminescence was performed with a Hitachi F4500 fluorescence spectrophotometer and a confocal Raman microscope with a 325-nm He-Cd laser. The absorption spectra were collected by a Shi-madzu UV-3101PC spectrometer. The Fourier transformed infrared (FTIR) was recorded with a Brucker VERTEX spectrometer; transmission electron microscopy (TEM) images were recorded on a FEI Tecnai G2 20S-twin. The dynamic light scattering (DLS) study was performed with a Malvern Zetasizer Nano ZS. X-ray diffraction (XRD) patterns were collected with a Bruker D8 system. The fluorescence decay profile was investigated using an Edinburgh FLS920 fluorescence spectrometer. Raman spectra were performed on LabRAM HR Evolution (Horiba) with a laser excitation at 532 nm. The X-ray photoelectron spectroscopy (XPS) analysis was measured by PHI 5000 Versa Probe (ULVAC-PHI, Japan). Atomic force microscopy (AFM) measurements were carried out with MultiMode scanning probe microscope (MM-SPM).

## Results and Discussion

**Fig. 1:**
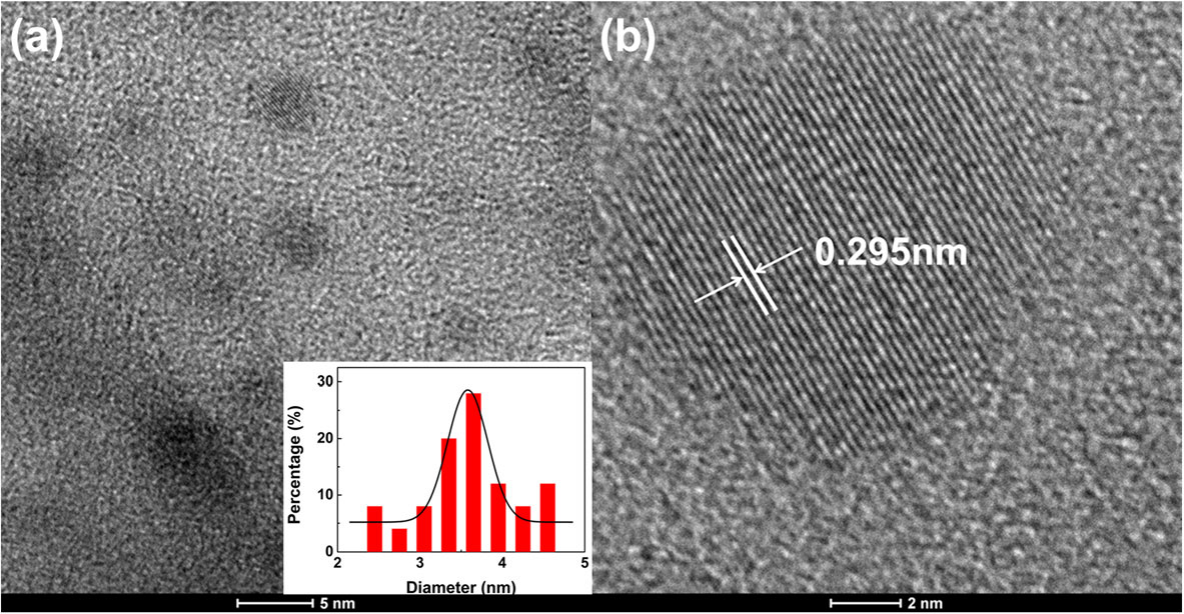
TEM and HRTEM images of as-prepared CDs. **a** The transmission electron microscopy (TEM) image of as-prepared CDs (*insets* showing particle-size distribution). **b** The high-resolution TEM image of one representative CD, which shows its crystallized graphite inner core

The formation of CDs is confirmed by transmission electron microscopy, X-ray diffraction (XRD) and Raman spectroscopy measurements. As shown in Fig. 1a, spherical carbon nanoparticles are obtained with an average diameter about 3.6 nm. The inset displays the distribution of particle sizes between 2.5 and 5 nm. Figure 1b shows that the CDs have crystallized inner cores with lattice spacing of 0.295 nm, which corresponds to the (002) plane of graphitic carbon [4, 9, 14]. Discernible lattice structures of CDs in the TEM images indicate that the resultant nanoparticles have the inner cores of graphite. The XRD diffraction pattern of the CDs shows a wide peak at 20.24° (Additional file 1: Figure S1), close to the (002) interlayer spacing of a graphitic structure [5, 21]. The G band at 1598 cm^−1^ and D band at 1350 cm^−1^ of CDs were not obvious on the Raman spectra (Additional file 1: Figure S2). The Raman characterization might be disturbed by the strong fluorescence of CDs. Also, the absence of the two peaks further proves that the CDs are composed of nanocrystalline graphite-like core and disordered sp^3^-carbon [21].

**Fig. 2:**
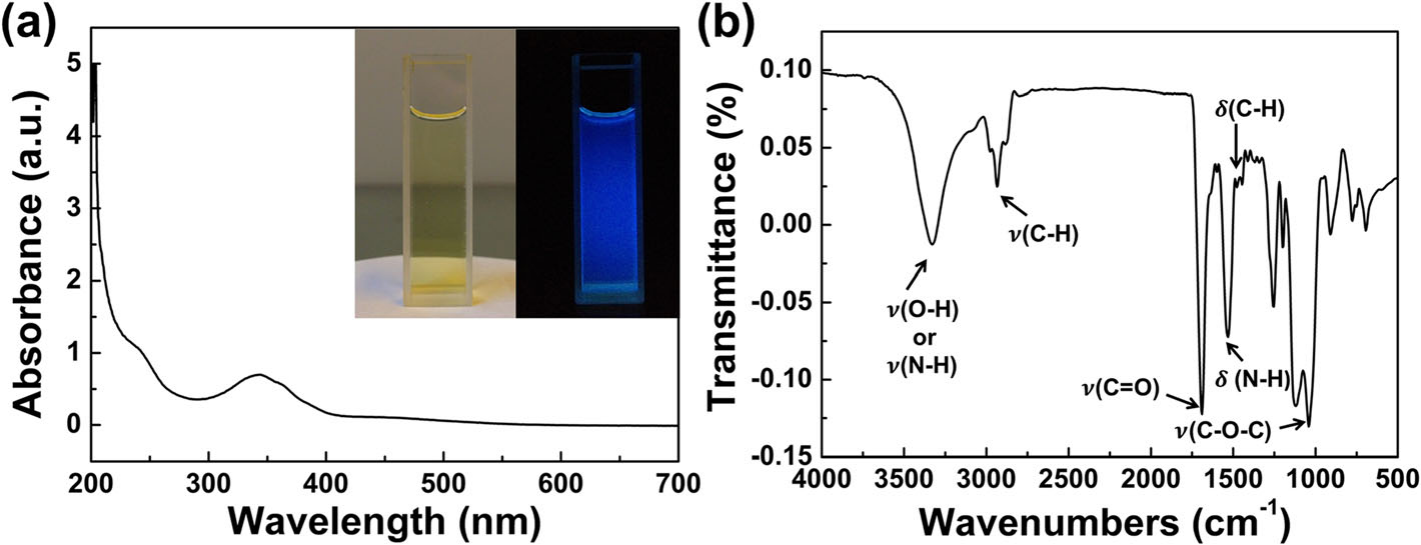
UV-vis absorption spectrum and FTIR spectrum of as-prepared CDs. **a** The UV-vis absorption spectrum of CDs. The *inset* photographs show the as-prepared CDs under natural light (*left*) and under 365 nm irradiation (*right*). **b** The FTIR spectrum of the CDs

As shown in Fig. 2a, the colour of as-prepared CD aqueous solution is yellowish (left), which exhibits the bright blue luminescence under the excitation of 365 nm UV light (right). In the absorption spectrum of CD solution, the absorption peak at 243 nm is attributed to $\pi$→$\pi$ ^*^ of C=C,and the absorption peak at 345 nm corresponds to n→$\pi$ ^*^ transition of the C=O bond (Fig. 2a) [14]. The Fourier-transform infrared spectroscopy (FTIR) spectrum (Fig. 2b) of the CDs suggests the presence of abundant oxygen-containing groups on their surfaces. As depicted in Fig. 2b, the peaks at 1120 cm^−1^ can be ascribed to the asymmetric and symmetric stretching vibrations of C–O–C. The peak at 1445 and 1464 cm^−1^ are assigned to C–H bending vibration; the peaks at 1488 cm^−1^ indicate the existence of N–H bending vibration; the peak at 1689 cm^−1^ is attributed to C=O stretching vibration; the peaks at 2935 cm^−1^ are arising from C−H stretching vibrations of methyl/methylene; and the broad band centered at 3100–3500 cm^−1^ are assigned to O–H and N–H stretching vibrations [5, 10, 14]. The results of FTIR analysis confirmed the presence of oxygen-containing groups on the surface of the as-prepared CDs, such as C = O and −OH. The XPS surveys further supported the FTIR analyses. As shown in the Additional file 1: Figure S3, the CDs are mainly composed of carbon, oxygen and nitrogen elements. The high-resolution XPS spectrum of C 1s show three peaks at 284.56, 285.66 and 287.7 eV, which indicate the presence of C=C/C–C, C–O and C=O. The high-resolution spectrum of N1s suggested the presence of pyrrolic-like N (399.7 eV) and graphitic-like/amino N (400.7 eV). The two peaks of O 1s high-resolution spectrum at 531.55 and 532.31 eV are attributed to C=O and C–OH/C–O–C bonds [21–24]. The results of XPS analysis is in good agreement with the FTIR spectrum. Combining all these characterization data, the CDs were considered to be composed of nanoscaled graphite-like core and oxygen-containing groups which on the surface of the core.

**Fig. 3:**
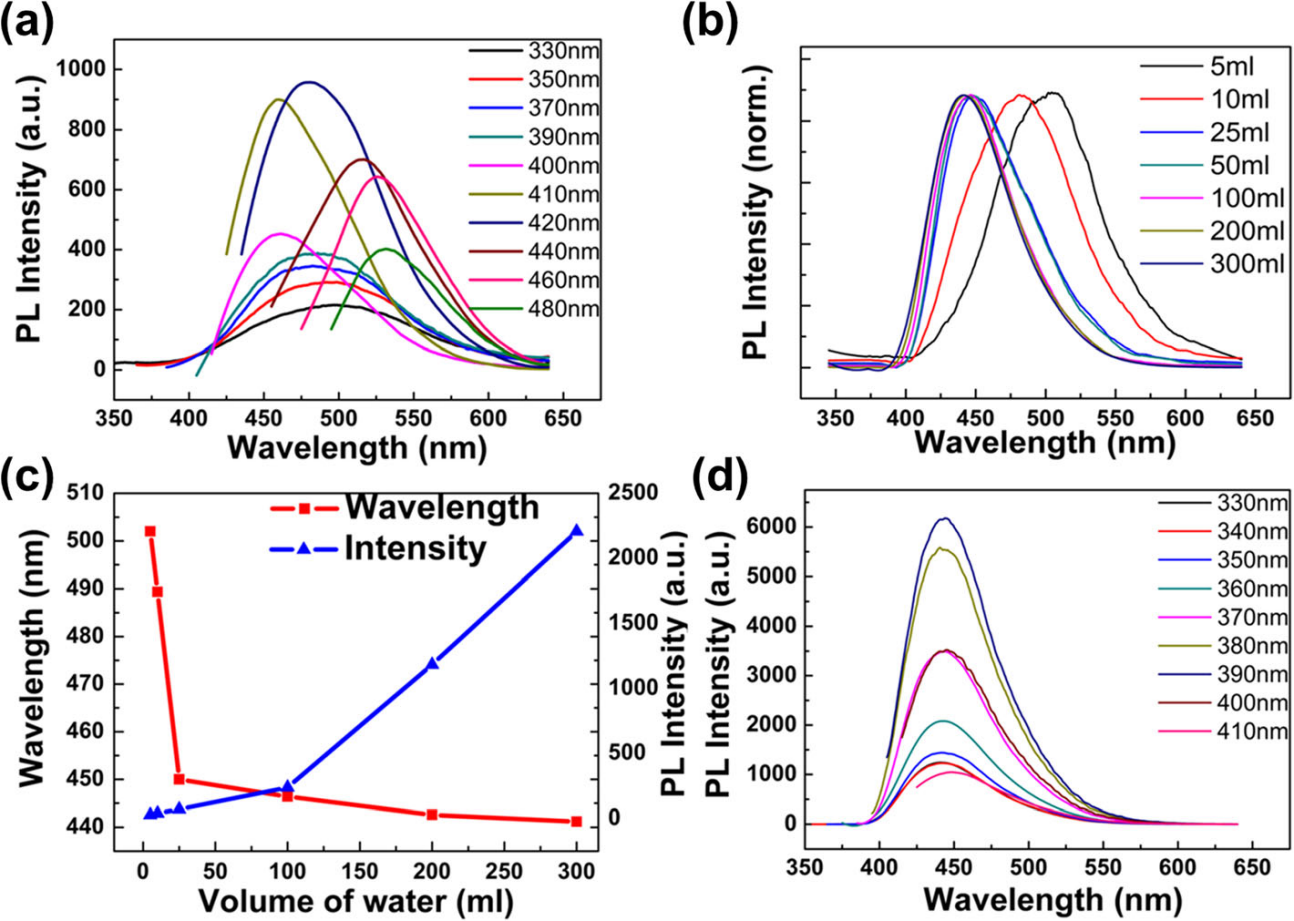
PL spectra of CDs in different volume of deionized water. **a** PL spectra of 1 ml as-prepared CDs with 5 ml deionized water (pH 10.41). **b** Normalized fluorescence emission spectra of CDs in different volume of water with the excitation wavelength of 330 nm. **c** The maximum PL intensity and the emission peak as the function of different volume of added water. **d** Emission spectra of diluting 1 ml as-prepared CDs solution with 300 ml deionized water

The emission spectra of CDs diluted by 5 ml deionized water show a typical excitation-dependent feature. The PL peak shifts to longer wavelength as the excitation wavelength gradually increases (Fig. 3a; the excitation wavelengths progressively increasing from 330 to 480 nm, and the emission intensity of 330–390 nm is multiplied by 25), which agrees well with other reports [1, 5, 14]. The CDs have a maximum emission intensity at 481 nm with an excitation of 420 nm. Using quinine bisulfate (QY 0.56 in 0.1 M H_2_SO_4_) as the reference, the quantum yield of the CDs is 74.8%. The high quantum yield is supposed to be the molecule state of CDs [5]. Moreover, both the emission wavelength and PL intensity of the obtained CDs are sensitive to the volume of added water; in other words, they are sensitive to the concentration of CD solution (Fig. 3b–d). This result is different from the CDs synthesized by other methods, which only showed a slight shift of the emission peak with the variation of pH value [25]. By adding more deionized water (10, 25, 50, 100, 200, 300, and 400 ml) into 1 ml as-prepared CD solution, the blue-shifts of the emission peaks are observed from 480 to 440 nm (Additional file 1: Figure S4), while the corresponding absorption spectra of CD solution has no change (Additional file 1: Figure S5). The intensity of emission peaks in the range of 330 to 400 nm are gradually enhanced, while the emission peaks in the range of 420 to 480 nm gradually disappear (Additional file 1: Figure S4). This blue-shift could be clearly seen in the normalized PL spectra in Fig. 3b, when the CDs with different concentrations are excited by the same wavelength of 330 nm. In Fig. 3b, c, the change of emission wavelength mainly occurs when the volume of added deionized water is less than 25 ml, which varies from 505 to 450 nm. With further dilution, the emission wavelength changes quite small. In Fig. 3c, the PL intensity of the emission peak continuously increases with the decreasing concentration of CDs. This intensity enhancement may benefit from the reduction of collisional quenching and self-absorption quenching in high-concentration solutions [5, 26].

After being diluted with 300 ml deionized water, the PL spectra show a single-emission peak at 443 nm, which is invariable as the excitation wavelength varies (Fig. 3d; the excitation wavelengths progressively increasing from 330 to 410 nm). The highest emission intensity is obtained under the excitation wavelength of 390 nm. Even when diluted with more volume of water (Additional file 1: Figure S4), the emission spectra are not shifted.

**Fig. 4:**
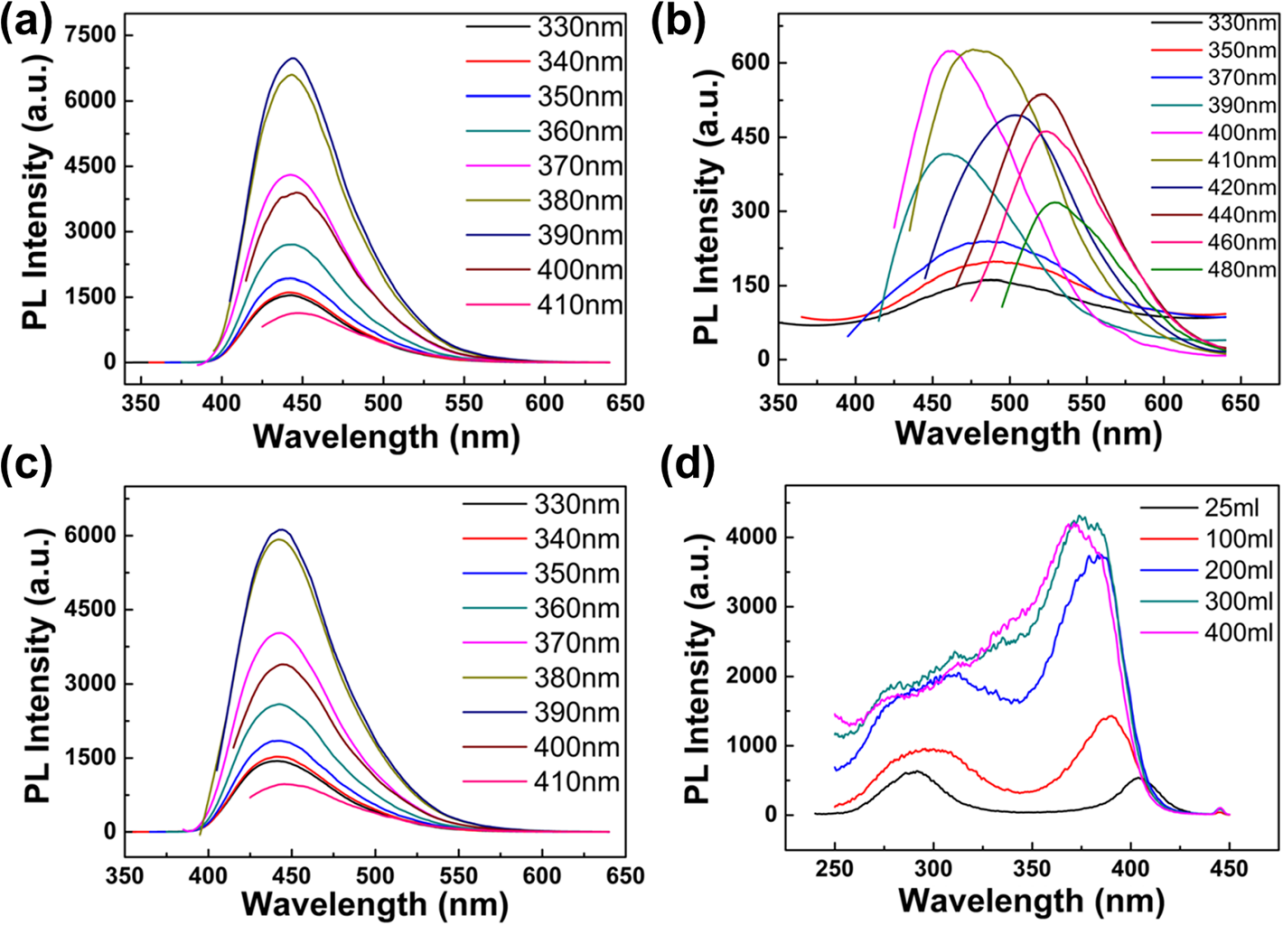
Emission and excitation spectra of CDs in different volume of water and pH. **a** Emission spectra of CDs with 300 ml deionized water (pH 10.41). **b** Emission spectra of CDs with 5 ml deionized water (pH 12.08). **c** Emission spectra of CDs with 300 ml deionized water (pH 12.08). **d** Excitation spectra of CDs at 445 nm in different volume of water

When adding different volumes of deionized water into as-prepared CD solutions, the pH value of the solution is changed. The phenomena we observed might be caused by the different pH values. In order to verify whether the pH value is the main cause of the phenomena, the CD solution of different pH values were analyzed in details. The pH value of the CD solution diluted by 5 ml deionized water is 10.41. When diluting the as-prepared CD solution with 300 ml deionized water, the pH value changes to 10.2. Then, we adjusted the pH value of the 300 ml diluted CD solution from 10.2 to 10.41 by adding NaOH. Figure 4a shows the PL spectra of the CD solution after adjusting the pH value to 10.41 (the excitation wavelengths progressively increasing from 330 to 410 nm). From the two figures (Figs. 3d and 4a), we could obviously notice that even when the pH value is adjusted from 10.2 to 10.41, the PL peak position and intensity are almost invariable. Then, we adjusted the pH values of the CD solution containing 5 ml deionized water and the solution containing 300 ml deionized water to the same value of 12.08 by adding NaOH (in Fig. 4b, the excitation wavelengths are progressively increasing from 330 to 480 nm, the emission intensity of 330–380 nm is multiplied by 15, and the emission intensity of 390 nm is multiplied by 6; in Fig. 4c, the excitation wavelengths are progressively increasing from 330 to 410 nm); compared with Fig. 3a,d, the positions and intensities of PL emissions also have no change. The above results show that pH is not the reason for the unchanging emission peak in our experiment. Thus, it can be concluded that concentration is the key point to adjust the emission wavelength and fix the emission peak. To our knowledge, this is the first report to show that the emission wavelength and PL intensity of CDs can be easily tuned by adjusting the concentration of CD solution with deionized water.

To provide insights into the excitation-independent characteristics of the CDs, we measured the excitation spectra of different emission wavelengths and showed it in Additional file 1: Figure S6. For CD solution with high concentration (1 ml as-prepared solution diluted with 25 ml deionized water), there are two strong excitation peaks located at 290 and 400 nm, separately. With the concentration decreasing from high to low, the excitation peak at 290 nm becomes weak and the peak at 400 nm is enhanced and blue-shifts to 370 nm (Fig. 4d). The characteristics of excitation spectra at different concentrations reveal that the luminescence of the CDs may have multiple centers. To make a further proof, the fluorescence lifetime of CDs (1 ml as-prepared solution diluted with 25 ml deionized water) was measured with the excitation wavelength in 280 nm and emission wavelengths in 447 nm (Additional file 1: Figure S7). The average lifetime is 11.85 ns, and the decay curve can be fitted by a double-exponential function with lifetimes of 5.11 ns (35.08%) and 13.28 ns (64.92%). The multiple lifetimes of the sample might be due to the diverse fluorophores or energy levels present on the surfaces of the samples [18].

Some studies of CDs showed the existence of small particles as well as particle aggregates even if a dilute solution was used [27]. A similar type of aggregation is also observed by Iijima [28], where small carbon particles are found to aggregate into 80-nm-size nanohorn structures. The small particles are attracted to each other by Van der Waals forces. We have estimated the particle sizes of CDs with different volumes of deionized water by the dynamic light scattering (DLS) measurement (Additional file 1: Figure S8), and the result shows that the hydrodynamic diameters of CDs are different, which ranges from 34 to 15 nm. In as-prepared CDs solution (high concentration), the average diameter is 34 nm. After diluting with 100 ml deionized water, the CDs show an average diameter of 15 nm. The average sizes of the CDs in aqueous solution have showed a decreasing trend with the decreasing concentration (Additional file 1: Figure S8). Accordingly, it can be concluded that when at high concentration, a number of single CDs have aggregated together to form the nanosized clusters, which lead to the increase of the average diameter. The single CDs and nanosized clusters are co-existent in the solution. While at low concentration, the nanosized clusters have been separated into single CDs. The average sizes of CDs tested by DLS measurement are larger than that of TEM results (4–6 nm), which is mainly because the DLS considers the overall hydrodynamic diameter that includes particles as well as absorbed molecules and ions [27]. The atomic force microscopy (AFM) of CDs with different volume of water was measured. As shown in Additional file 1: Figure S9, when the concentration is high, the image reveals that the single CDs have aggregated together to form the nanosized clusters, and the average diameter is 40 nm; as the concentration decreases from high to low, the nanosized clusters gradually separated into single CDs, and the measured diameter is about 10 nm which is smaller than 40 nm, which is in good consistence with the result of DLS.

The formation of CDs from organic materials in aqueous media has a common viewpoint that the CDs are composed of nanocrystalline cores of sp^2^-hybridized two-dimensional graphene-type islands [10, 29] disrupted by sp^3^-hybridized diamond-type inclusions [27, 29]. During the formation of nanoparticles, the polar groups derived from starting materials are attached on the surface of CDs, which allows the particles to be soluble in water. This viewpoint were confirmed by the Raman spectra of the CDs obtained from different starting materials [27, 30], which demonstrated the presence of sp^2^- and sp^3^-hybridized structures in similar proportions. Meanwhile, all studied water-soluble CDs obtained by the thermal treatment of organic materials contain oxygen elements in the form of hydroxyls, carboxyls and carbonyls [16]. The polar groups on the particle surface are of particular importance for the emission of CDs [16, 18, 31].

**Fig. 5:**
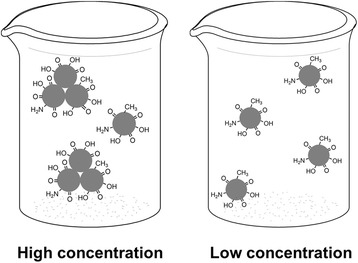
The sketch of CDs at high concentration and low concentration. *Left*: when at high concentration, a number of single CD formed nanosized clusters; *right*: when at low concentration, the nanosized clusters separated into single CDs

From the above experiment, we can conclude that there are two different emissive species in CD solution. The intrinsic luminescence from sp^2^-carbon networks and the high polarity of nanosized clusters might contribute to the different emission phenomenon (Fig. 5). The single CD is found to behave like an electric dipole [32] due to its polar surface groups, such as –CO and –OH [15, 18, 31] (Fig. 2b). The oxygen-containing groups on the surface of the CDs can be responsible for the longer wavelength part of fluorescence emission [19]. When at high concentration, a number of CDs are aggregated together by Van der Waals forces [28] to form nanoclusters, then a large number of –CO and –OH get together, which leads to the higher polarity on the surfaces of nanoclusters [15]. The high polarity of the nanosized clusters causes the properties of excitation-dependent [15, 19, 31]. Meanwhile, the high degree of oxidation and higher polarity of the nanosized clusters lead to electron rapid relaxation from excited states to substates, which corresponds to longer wavelength. Then, the substates contribute to photo emission, which eventually gives rise to longer wavelength emission [15]. So, the phenomenon of excitation-dependent was occurred at longer wavelength part when at high concentration. After adding deionized water into the as-prepared CD solution, the concentration of the solution gradually decreased. Then, CDs that formed nanoclusters are separated and re-dispersed into single CDs, which leads to the weakening of the polarity and the disappearance of emission spectra at longer wavelength. Furthermore, the separation of the clusters also leads to the disappearance of excitation peak at 290 nm (Fig. 4d). Compared with the emission from the polar groups (–CO and –OH) on the surface of single CD, the intrinsic luminescence from sp^2^-carbon networks plays a dominate role with the decrease of CD concentration. When at low concentration, the fluorescent spectrum of single CD with only intrinsic luminescence is asymmetric and broadened to the higher energy region (short wavelength), which shows the excitation-independent fluorescence as shown in Fig. 4a [15, 33].

## Conclusions

In summary, we have synthesized the CDs by hydrothermal method. Significantly, the as-prepared CDs presented excellent aqueous dispersibility and unique PL properties such as concentration-sensitivity and excitation-independent emission wavelength. Blue-shifts of the maximum emission wavelength from 480 to 443 nm were observed when the concentration of CDs decreased from high to low. The PL spectra at low concentration of CDs showed an excitation-independent behaviour with the emission peak at 440 nm, which is very different from the previous reports. It could be concluded that there are two different emitting mechanisms. The intrinsic luminescence from sp^2^-carbon networks was responsible for the emission at short wavelengths (excitation-independent) at low concentration, and the high polarity of nanosized clusters led to the excitation-dependent property of the longer wavelength part when at high concentration. The favourable photophysical properties and concentration-dependent behaviour of the CDs will provide a way to tune the emission wavelength and offer new insights into CDs from the viewpoints of both experiments and mechanisms, which will promote diverse potential applications of CDs in the near future.

## Additional file

Additional file 1: The XRD diffraction pattern of the CDs shows a wide peak at 20.24°. (DOCX 5972 kb)

## Abbreviations

AFM: Atomic force microscopy
CDs: Carbon dots
DLS: Dynamic light scattering
FTIR: Fourier transformed infrared
PL: Photoluminescence
TEM: Transmission electron microscopy
XPS: X-ray photoelectron spectroscopy
XRD: X-ray diffraction
