# Supplementary material for: High-Efficient Excitation-Independent Blue Luminescent Carbon Dots
Source: Nanoscale Res Lett. 2017 Jun 10;12:399. doi: 10.1186/s11671-017-2137-2 (PMC5466853; doi:10.1186/s11671-017-2137-2)
Supplement: Additional file 1: — The XRD diffraction pattern of the CDs shows a wide peak at 20.24°. (DOCX 5972 kb) [file 11671_2017_2137_MOESM1_ESM.docx]

Supporting information

High efficient excitation-independent blue luminescent carbon dots

Hongzhen Liu^1,2†^, Xin Zhao^1,2†^, Fei Wang^1^, Yunpeng Wang^1^*, Liang Guo^3^, Jingjing Mei^1,2^, Cancan Tian^1,2^, Xiaotian Yang^3^, and Dongxu Zhao^1^*

*^1^State Key Laboratory of Luminescence and Applications, Changchun Institute of Optics, Fine Mechanics and Physics, Chinese Academy of Sciences, No. 3888 Dongnanhu Road, Changchun, 130033, People’s Republic of China.*

*^2^University of Chinese Academy of Sciences, Beijing, 1000493*

*^3^Jilin Provincial Key Laboratory of Architectural Electricity & Comprehensive Energy Saving, Jilin Jianzhu University, Changchun, 130118,People’s Republic of China.*

**E-mail:* [*wangyunpeng@ciomp.ac.cn*](mailto:wangyunpeng@ciomp.ac.cn)*;* [*zhaodx@ciomp.ac.cn*](mailto:zhaodx@ciomp.ac.cn)

*^†^These authors contributed equally to this work.*

**Quantum yields (QY) measurements:**

The quantum yield of the C-dots was measured using quinine sulfate as the reference material, and calculated with following equation:

Q$Y_{X}$=Q$Y_{\mathrm{std}}$ (${I_{X}}/{I_{\mathrm{std}}}$)/ ($A_{std}$/$A_{X}$)(${\eta_{X}^{2}}/{\eta_{\mathrm{std}}^{2}}$)

where the subscript “x” designates the C-dots, the subscript “std” designates quinine sulfate, “QY” stands for the quantum yield, “I” stands for the integrated PL intensity, “A” stands for the absorbance, and “$\eta$” stands for the refractive index of the solvent. Quinine sulfate (quantum yield: 56%) was dissolved in 0.1 M H_2_SO_4_ (refractive index: 1.33) and the C-dots were dissolved in water (refractive index: 1.33). In order to minimize reabsorption effects, absorbance value of the individual solution was kept below 0.10 at the excitation wavelength (350 nm).


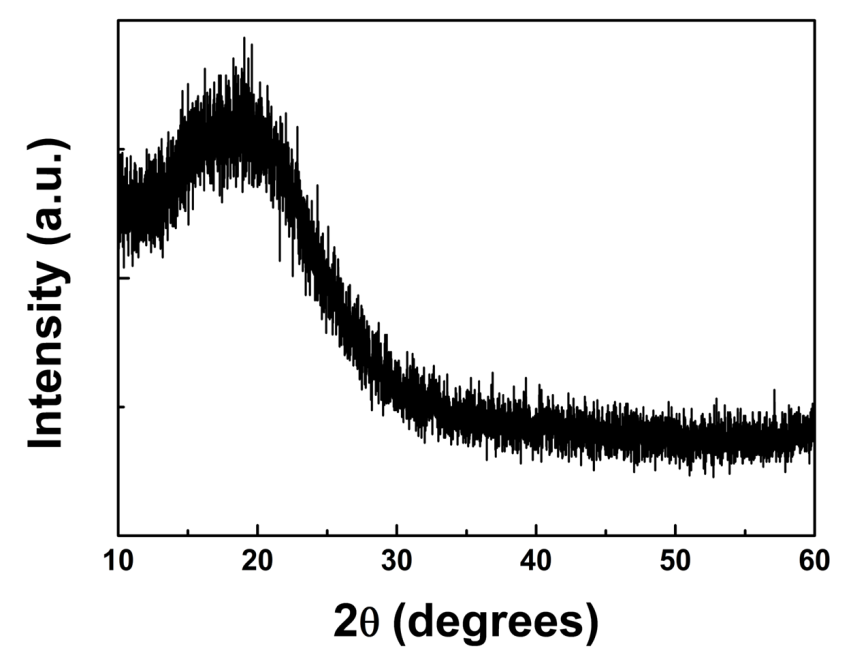


**Figure** **S1.** XRD diffraction pattern of the as-prepared CDs


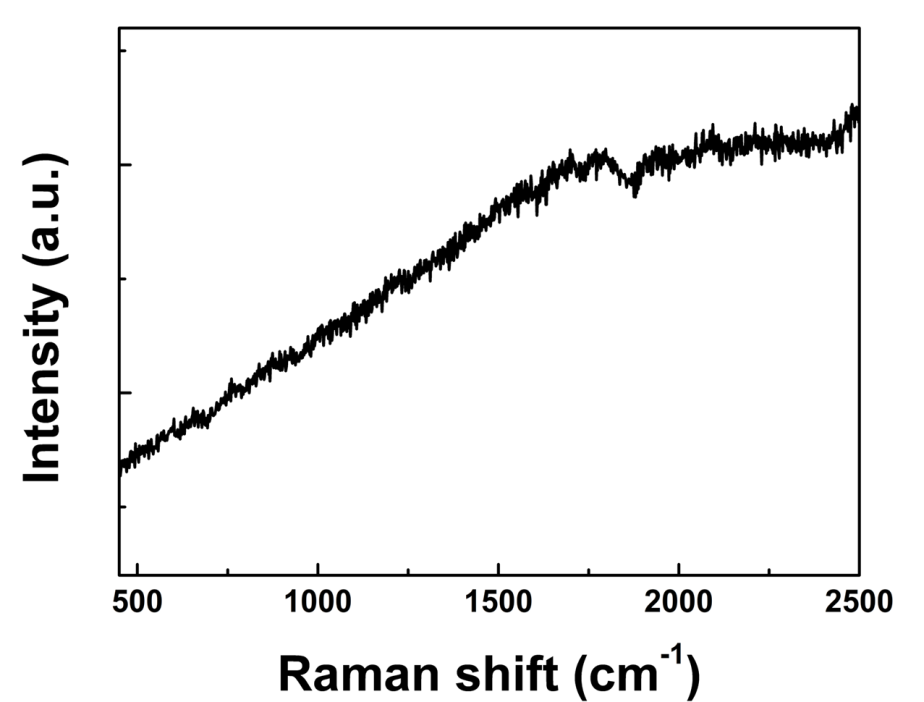


**Figure** **S2.** Raman spectrum of the as-prepared CDs


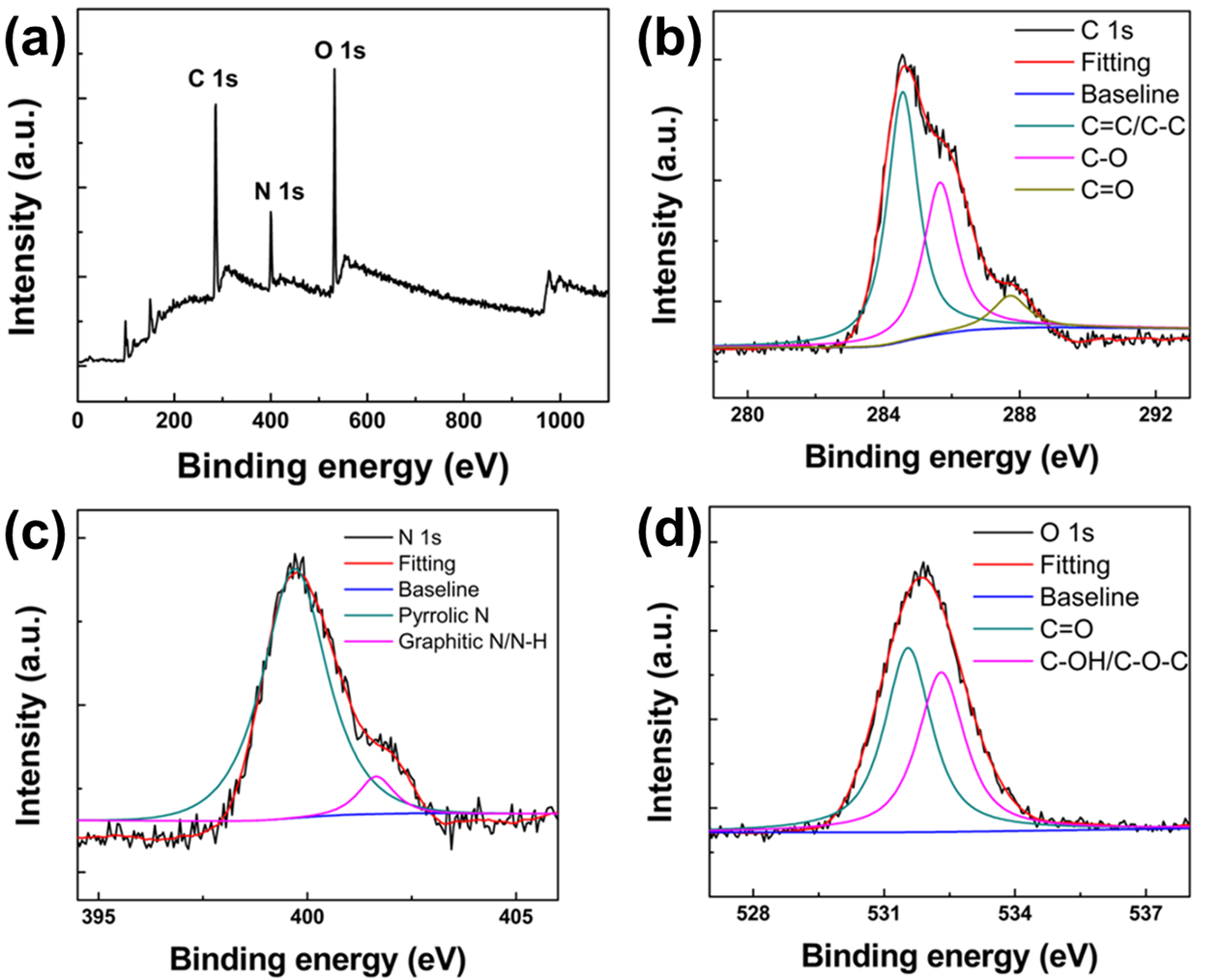


**Figure** **S3.** (a) Wide scan XPS survey; (b) C 1s XPS spectra; (c) N 1s XPS spectra; (d) O1s spectra of the as-prepared CDs.


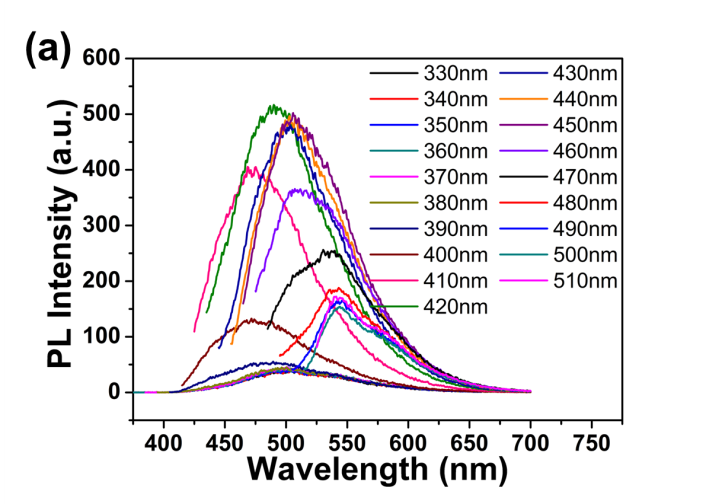

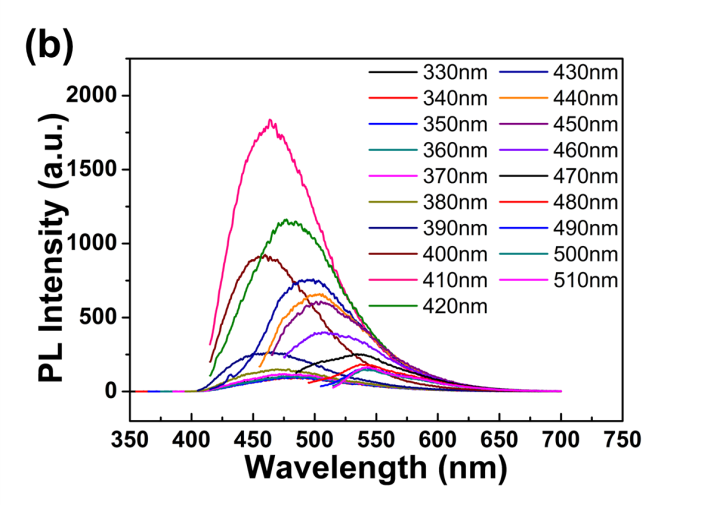


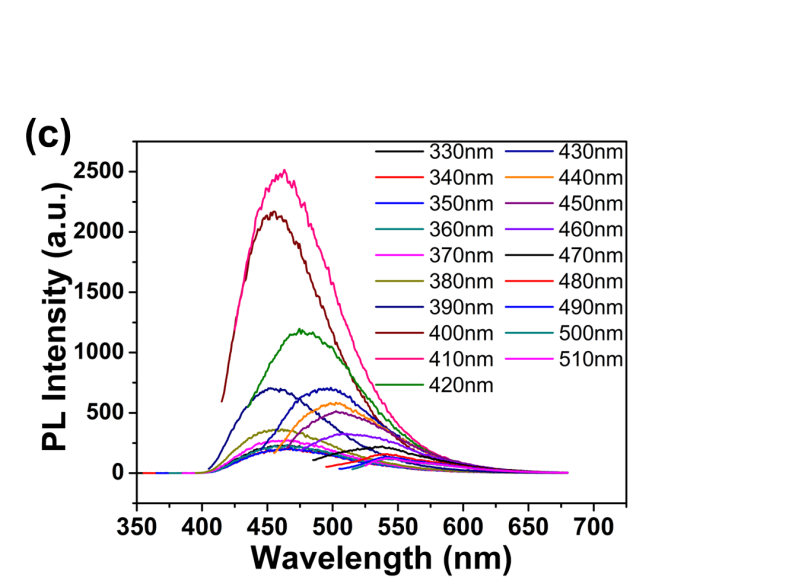

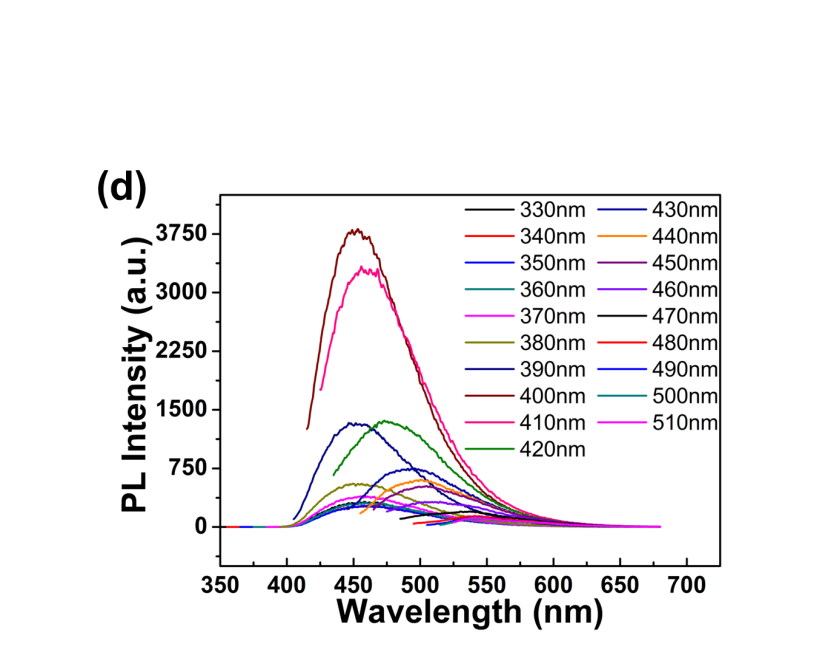


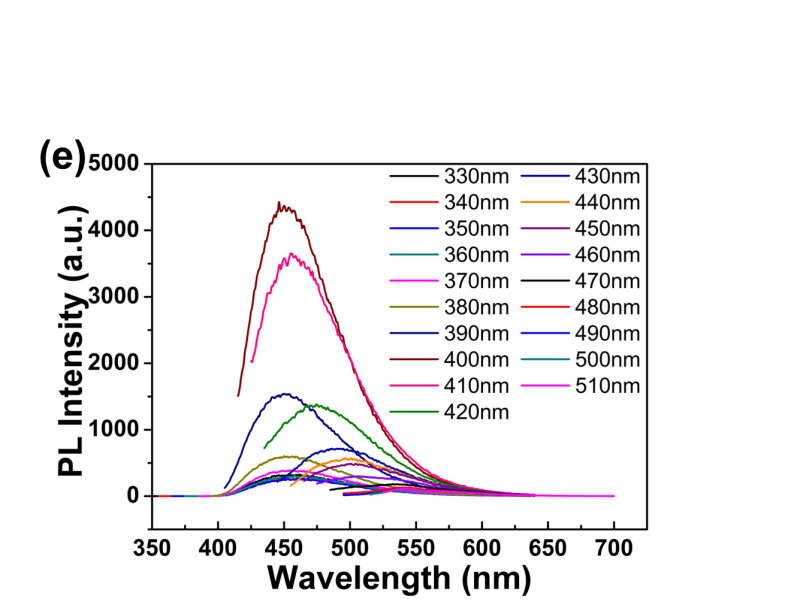

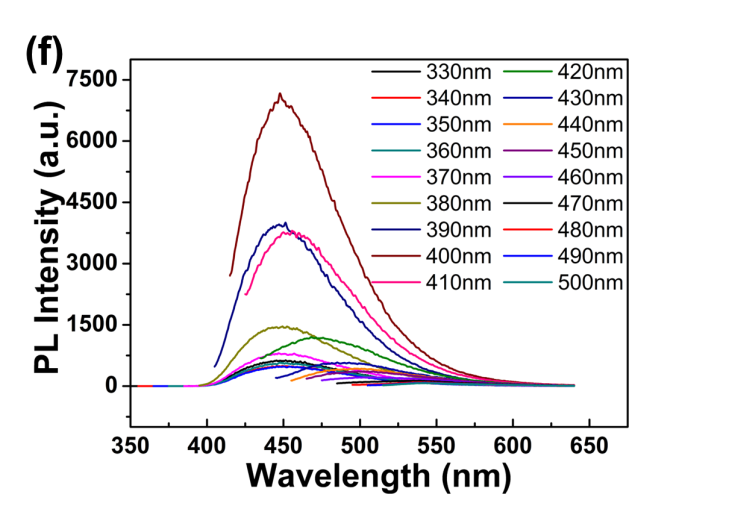


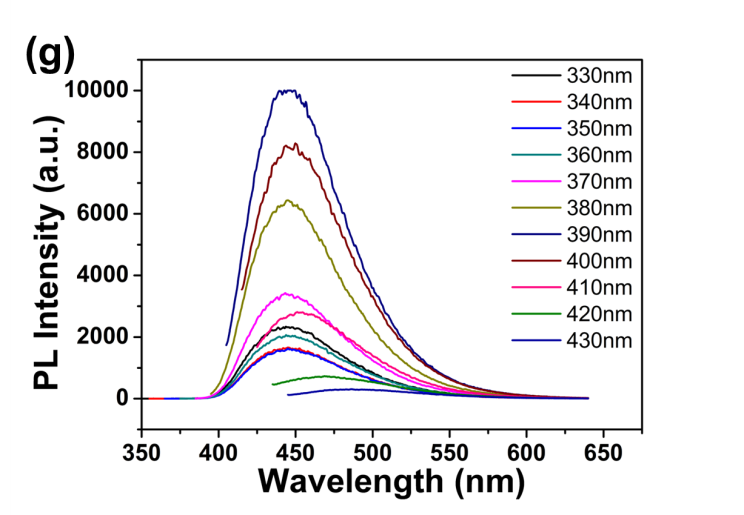

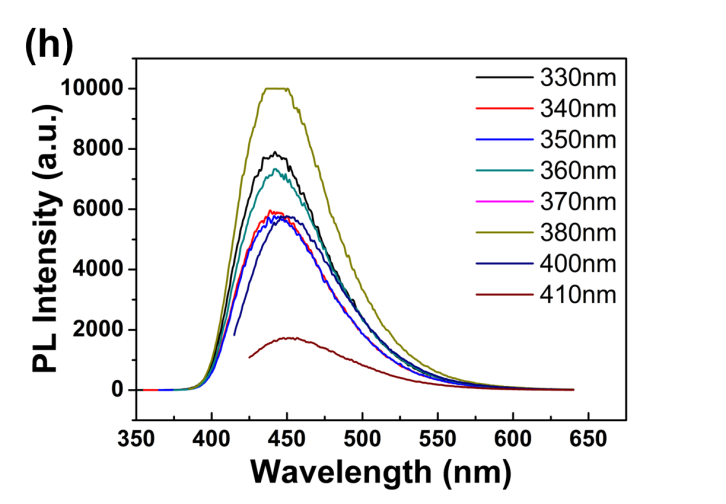


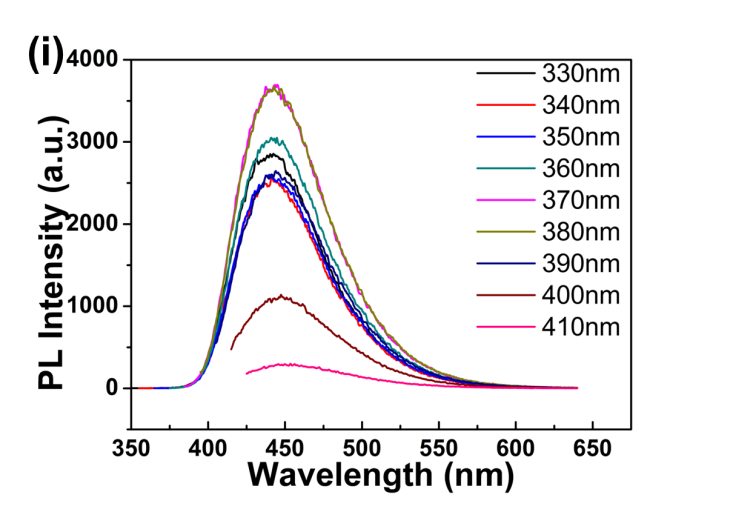

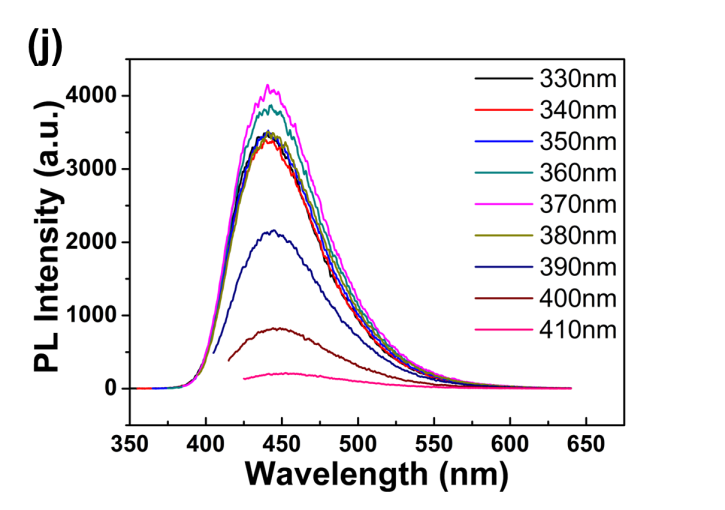


**Figure** **S4.** PL emission spectra of CDs with different volume of water (a: 5 ml; b: 10 ml; c: 15 ml; d: 20 ml; e: 25 ml; f: 50 ml; g: 100 ml; h: 200 ml; i: 300 ml; j: 400 ml) under different excitation wavelengths. As the concentration of CDs decreases from high to low, the excitation-dependent behavior is vanished.


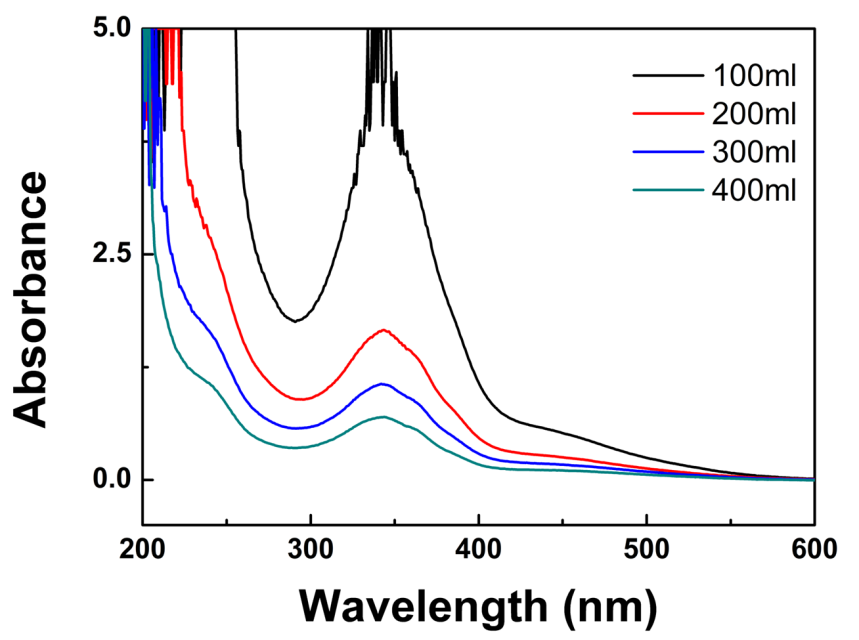


**Figure** **S5.** The UV-vis absorption spectrum of CDs in different volume of water


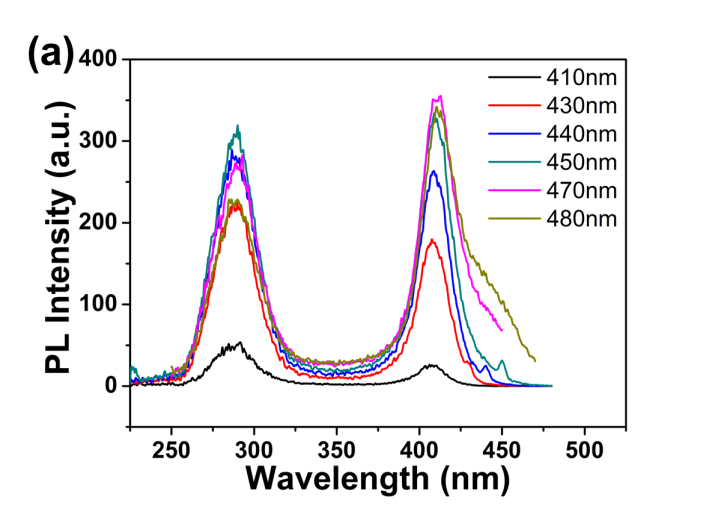

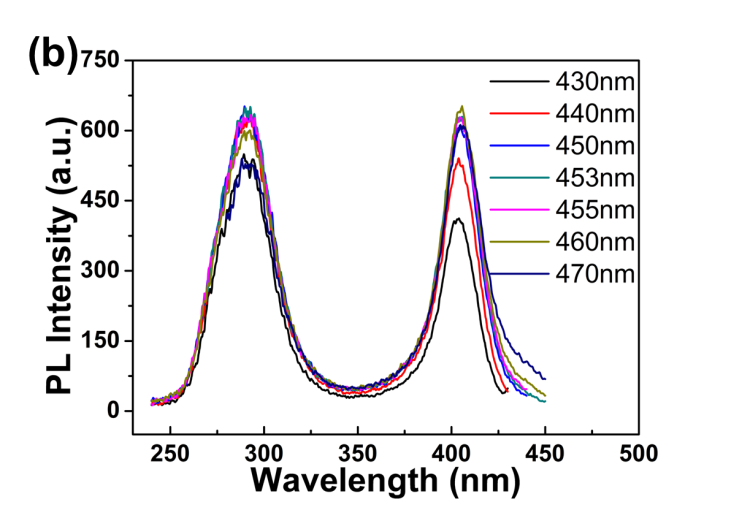


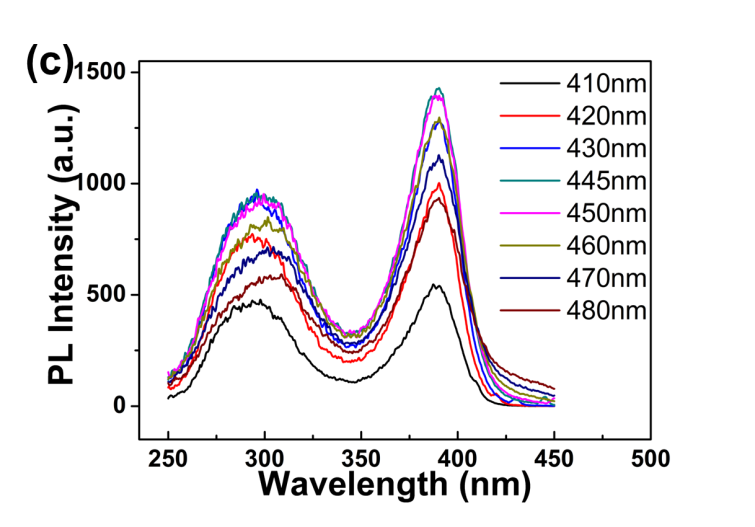

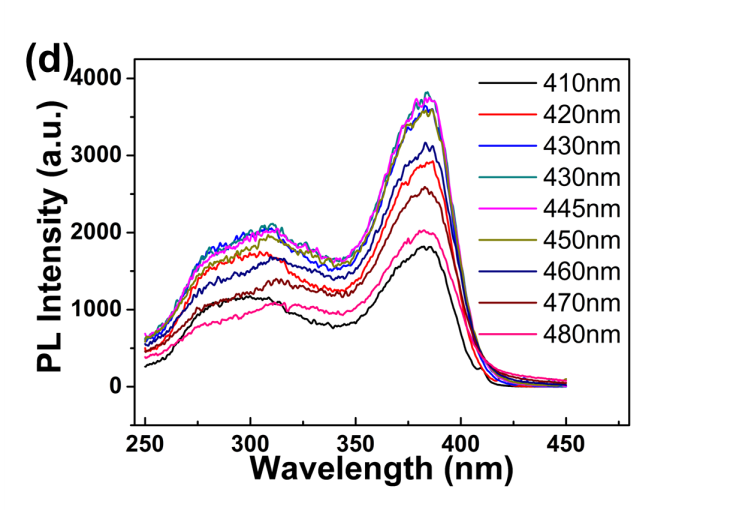


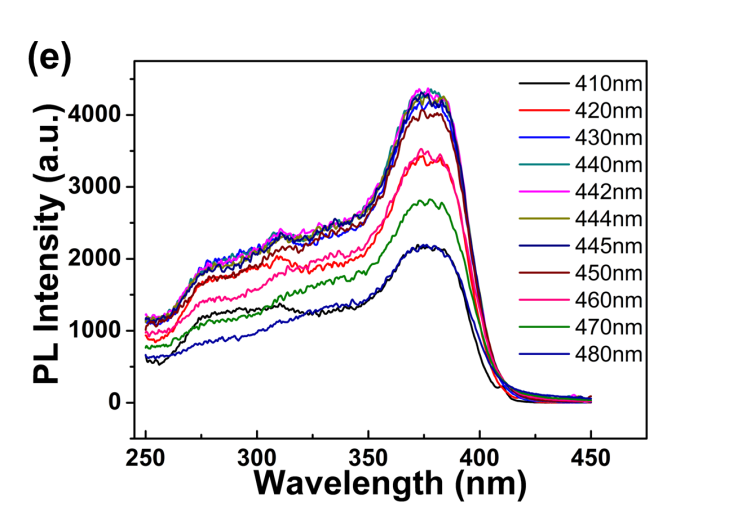

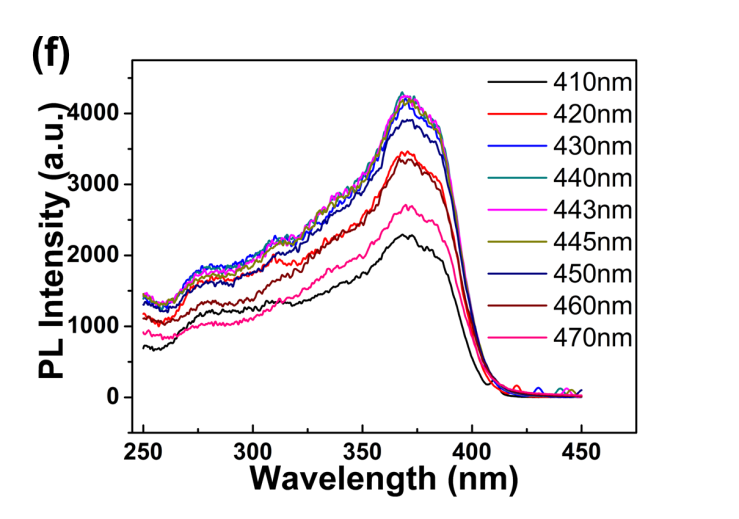


**Figure S6.** PL excitation spectra of CDs in different volume of water (a: 10 ml; b: 25 ml; c: 100 ml; d: 200 ml; e: 300 ml; f: 400 ml). When at high concentration, the excitation spectra show two strong excitation peaks at 290 nm and 400 nm. As the concentration decreases from high to low, the excitation peak at 290 nm has disappeared, the peak at 400 nm becomes stronger and blue-shifts to 370 nm.


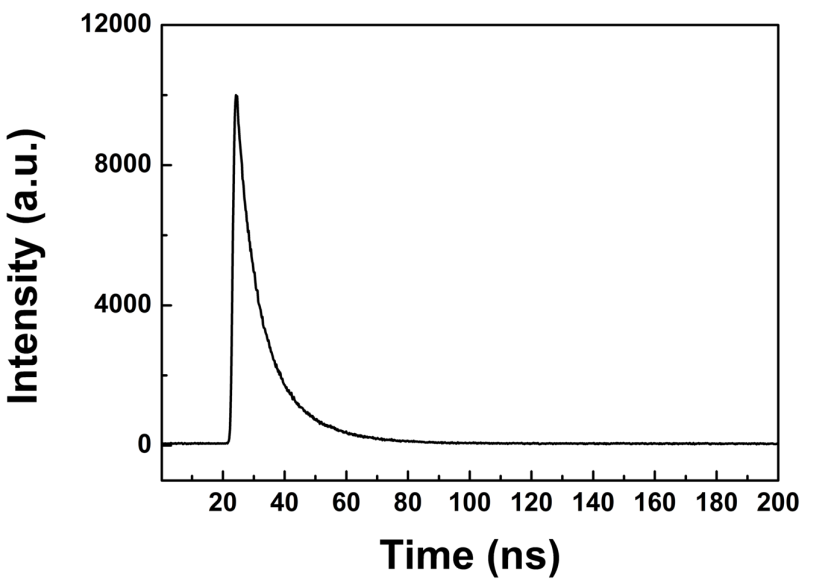


**Figure** **S7.** Fluorescence decay profile of CDs


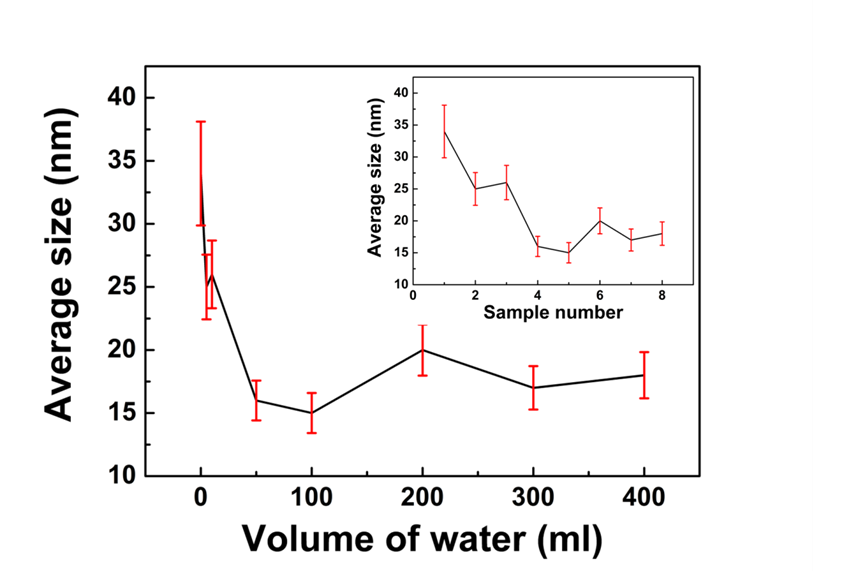


**Figure S8.** Average sizes of CDs in different volume of water; Inset: Average sizes of CDs in different volume of water sampled with different numbers (1: the as-prepared CDs solution; 2: 5 ml; 3: 10 ml; 4: 50 ml; 5: 100 ml; 6: 200 ml; 7: 300 ml; 8:400 ml. the corresponding PDI was: 0.212, 0.163, 0.175, 0.189, 0.185, 0.190, 0.177, 0.197 respectively)


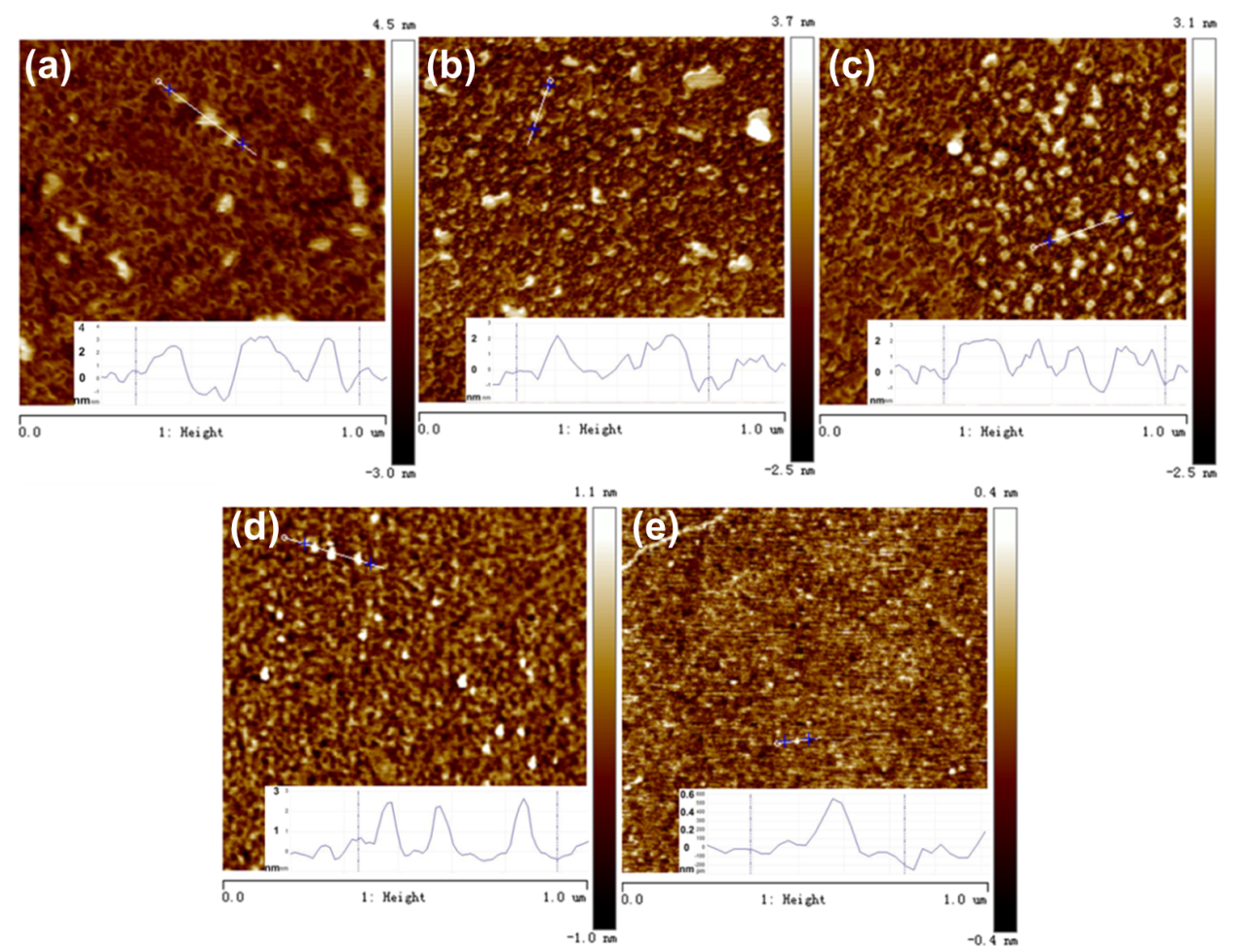


**Figure S9.** AFM image (inset showing height profiles analysis) of CDs with different volume of water (a: 5 ml; b: 50 ml; c: 100ml; d: 200 ml; e: 400 ml).
